# Prosodic Parallelism—Comparing Spoken and Written Language

**DOI:** 10.3389/fpsyg.2016.01598

**Published:** 2016-10-19

**Authors:** Richard Wiese

**Affiliations:** Department of Germanic Linguistics, Philipps-Universität MarburgMarburg, Germany

**Keywords:** prosody, German language, language processing, speech, writing

## Abstract

The *Prosodic Parallelism* hypothesis claims adjacent prosodic categories to prefer identical branching of internal adjacent constituents. According to Wiese and Speyer (2015), this preference implies feet contained in the same phonological phrase to display either binary or unary branching, but not different types of branching. The seemingly free schwa-zero alternations at the end of some words in German make it possible to test this hypothesis. The hypothesis was successfully tested by conducting a corpus study which used large-scale bodies of written German. As some open questions remain, and as it is unclear whether Prosodic Parallelism is valid for the spoken modality as well, the present study extends this inquiry to spoken German. As in the previous study, the results of a corpus analysis recruiting a variety of linguistic constructions are presented. The Prosodic Parallelism hypothesis can be demonstrated to be valid for spoken German as well as for written German. The paper thus contributes to the question whether prosodic preferences are similar between the spoken and written modes of a language. Some consequences of the results for the production of language are discussed.

## Introduction

### Prosody in written and spoken language

Language is manifested in a spoken and in a written modality (if ignoring sign language which has interesting aspects of prosody in itself, see e.g., Nespor and Sandler, 1999; Sandler et al., 2005). Just as segmental phonology concerns the phoneme-based organization of speech, prosodic phonology covers the non-segmental and suprasegmental organization of speech. Prosody thus consists in the hierarchical chunking of basic sound units (syllables build groups over segments) or suprasegmental properties such as tone and intonation. In their influential model, Nespor and Vogel (1986, p. 3) characterize prosody as “the mental representation of speech.” This definition emphasizes the cognitive status of prosody, and demonstrates, at the same time, that prosody is usually considered in relation to language in the spoken modality alone.

But this view is likely to be overly naive: prosody may well extend to language in the written modality. In fact, there is considerable evidence that prosodic wellformedness principles extend to written language: As shown by Ashby and Clifton (2005), Ashby (2006), and others, reading English written texts is guided by prosodic units. The existence of so-called “silent prosody” as a component of the processing of written texts has also been demonstrated by Fodor (2002) and Kentner and Vasishth (2016). Furthermore, grammatical variation in English has been shown to be subject to prosodic preferences. This is true, for example, for the so-called dative alternation (*give Mary a book vs. give a book to Mary*; see Anttila et al., 2010), and the alternation in the genitive construction (*Germany's government* vs. *the government of Germany*; see Schlüter, 2005; Shih et al., 2015). Punctuation is another area of written language which has been associated with prosody; see Chafe (1988). For German and other languages, there has been relatively little evidence of this sort so far; though see Kentner (unpublished manuscript). The present study constitutes another attempt to find evidence for the role of prosody in the organization of spoken utterances, and is based on the German language.

### Parallelism in written german

The observation that present-day German shows a remarkably high number of lexical items with a schwa-zero alternation at the end of words provides the starting point of the present study. Some cases of such alternating forms across different word classes are given in (1). Final <e> in the German orthography is usually pronounced [ә], the central short vowel called *schwa*, which is always unstressed. The respective syllable thus forms the weak syllable within a foot.

(1) Schwa-/zero alternations
  - nouns: Tür(e) ‘door’, Aug(e) ‘eye’, Ruh(e) ‘quietness’
  - inflected nouns, gen. sg.: Sturm(e)s, ‘storm’, Jahr(e)s ‘year’
  - adjectives/adverbs: bös(e) ‘bad’, bang(e) ‘afraid’, nah(e) ‘near’, gern(e) ‘gladly’, heut(e) ‘today’
  - verbs: (1st ps. sg.): hab(e) ‘have, present tense’, seh(e) ‘see, present tense’
  - prepositions: ohn(e) ‘without’

This list of cases is by no means complete, and under constraints from poetic meter, any word-final schwa^1^ can be dropped. For many of these cases, the two competing forms have different frequencies and/or are assigned to specific registers, regional dialects or style levels, but sometimes no such difference can be detected. For example, both *gern* and *gerne* ‘gladly’ occur with high frequency across Standard German variants, although the latter form was found to be twice as frequent in written German by Wiese and Speyer (2015). In the database of spoken German introduced below, the two forms display virtually identical frequencies: 2133 and 2017 tokens, respectively.

This raises the question whether such schwa-zero alternations, if not part of sociolinguistic or dialectal register variation, instantiate free variation. In the existing literature (Dudenredaktion, 1998; Szczepaniak, 2010; Fehringer, 2011), a few factors governing the alternation have been discussed (such as type of final pre-schwa segment, morphology, frequency). But it is unclear why the alternation exists at all, as many descriptions of prosody in German (e.g., Eisenberg, 1991; Féry, 1997; Wiese, 2009; Kentner, unpublished manuscript) have stressed the strong preference for the word-final bisyllabic trochaic foot (strong syllable—weak syllable), which should favor, for example, *gerne* over *gern* across the board. The existence of such alternations may also be related to the fact that the vowel schwa is unclear as to its status as a phoneme. Some authors (Wurzel, 1970; Wiese, 2009) have argued that schwa is non-distinctive because it is largely predictable from its consonantal context. Furthermore, morphological descriptions of German, such as Fleischer and Barz (1995), have pointed out that it is unclear whether final schwa constitutes a suffix or not. Given the optionality of final schwa just noted, it is likely that it is not a suffix because suffixes in general are obligatory. For example, while -e may be left out in final position as in *laufe/lauf* ‘run, present tense, 1st ps. sing.’, such an omission would be impossible for any other suffix in verbal inflection.

Wiese and Speyer (2015) propose the hypothesis that schwa in German may be regulated by a principle of “Prosodic Parallelism.” Prosodic Parallelism favors two adjacent feet from two adjacent phonological words within a single phonological phrase to display identical branching, either both unary or both binary. In other words, a phrase should consist either of two bisyllabic feet or two monosyllabic feet. Schwa-zero alternations such as those in (1) exist because they help to achieve parallelism. (2) Provides an illustration of this claim with the first example from (1): of the logically possible combinations with the monosyllabic definite and the bisyllabic indefinite determiner (nominative singular for feminine nouns) the ones achieving Prosodic Parallelism (bolded cells in (2)) should be preferred.

(2) Determiner noun combinations

**Table:** 

		Determiner form	
		Monosyllabic	Bisyllabic
Noun form	monosyllabic	**die Tür**	eine Tür
	bisyllabic	die Türe	**eine Türe**

More precisely, given the independently existing frequencies of the determiner forms and the alternating noun forms (for example, *Tür* happens to be much more frequent than *Türe*), the frequencies of the preferred combinations should be higher than expected from the combination of the frequencies of the two parts. Formulating the hypothesis in this way, it is testable, because present-day corpora of German contain the required combinations of words in sufficiently large numbers. Note that the combination of the noun with the two determiners provides the kind of minimal pair needed for the test of the hypothesis: there are two surface phrases with very similar meaning. All cases discussed below share this property.

Wiese and Speyer (2015) drew upon the DeReKo database (Deutsches Referenzkorpus/ Archiv der Korpora geschriebener, Gegenwartssprache, 2011), containing mostly newspaper texts, for a comprehensive test of the Prosodic Parallelism hypothesis. They made use of the existence of 15 relevant combinations of words displaying the schwa-zero alternation, covering the examples presented in (1) and some more. The study tested the hypothesis for each of the combinations by means of a series of chi-square tests and demonstrated that, in general, the phrases with uniform branching occurred with higher-than-chance frequencies. This was true for the case of *Tür(e)* as presented in (2) as well as for a large range of other words and phrases summarized in (3).

(3) Statistical tests for Prosodic Parallelism; results from Wiese and Speyer (2015)

**Table:** 

1	*die, der/eine, einer + Tür(e)* ‘the/a door’	$\chi_{\left(1\right)}^{2}$ = 14.784, *p* < 0.00012
2	*der/guter + Hirt(e)* ‘the/good shepherd	$\chi_{\left(1\right)}^{2}$ = 7.822, *p* < 0.005
3	*dem/einem + Tag(e)* ‘the/a day’	$\chi_{\left(1\right)}^{2}$ = 343.905, *p* = 0.00
4	*des/eines + Tag(e)s* ‘the/a day’	$\chi_{\left(1\right)}^{2}$ = 167.34; *p* < 0.0001
5	*des/eines + Jahr(e)s* ‘the/a year’	$\chi_{\left(1\right)}^{2}$ = 70.946, *p* < 0.001
6	*des/eines + Stier(e)s* ‘the/a bull’	$\chi_{\left(1\right)}^{2}$ = 28.038, *p* < 0.001
7	*gern(e)* ‘gladly’ vs. prefixed form *ungern(e)* ‘not gladly’	$\chi_{\left(1\right)}^{2}$ = 21988.526, *p* = 0.000
8	*nah(e)* ‘near’ with preceding verbs	$\chi_{\left(1\right)}^{2}$ = 131.335, *p* < 0.001
9	*nah(e)* ‘near’ with following verbs	$\chi_{\left(1\right)}^{2}$ = 7.474, *p* < 0.0063
10	*heut(e)* ‘today’ with following adverb	$\chi_{\left(1\right)}^{2}$ = 10.457, *p* < 0.001222
11	*wär(e) + gern(e)* ‘would gladly’	$\chi_{\left(1\right)}^{2}$ = 60.793, *p* = 0.000
12	*sehr/richtig + gern(e)* ‘very/really gladly’	$\chi_{\left(1\right)}^{2}$ = 55.712, *p* = 0.000
13	Set of 162 strong nouns in genitive singular form	Generalized linear model: trochaic nouns are more frequent with trochaic determiners
14	Stem-final schwa in nouns from Early New High German	$\chi_{\left(1\right)}^{2}$ = 1.68, *p* < 0.19494
15	Set of strong nouns in genitive singular from Early New High German	$\chi_{\left(1\right)}^{2}$ = 5.392, *p* < 0.02023

As shown here, the hypothesis could not be supported for four out of the 15 cases. For three of them, *des/eines Jahr(e)s, dem/einem Tag(e)*, and *sehr/richtig gern(e)*, the significant effects actually pointed into the direction opposite to what was predicted. One reason for these counter-examples might be that the prosodic phrasing is actually not the one assumed in the paper: dative noun phrases as in (3) predominantly appear within prepositional phrases, for which the prosodic phrasing may be as in *[[an dem] [Tag(e)]]* ‘at the day’. For more discussion of these real or apparent counter-examples see Wiese and Speyer (2015) and Kentner (2015).

## A study on parallelism in spoken german

As noted above, the Prosodic Parallelism hypothesis was tested by Wiese and Speyer (2015) on the basis of a corpus of *written* German. The reason for this choice was primarily a pragmatic one: written language corpora are much more comprehensive than those for spoken language. While Wiese and Speyer (2015) argue that the existence of Prosodic Parallelism in written language (where prosody possibly plays a less prominent role than it does in speech) makes the case for the hypothesis even stronger, Kentner (2015) suggests the possibility that Prosodic Parallelism (just like other prosodic preferences) may well be a specific property of written texts not to be extendable to the spoken modality. According to this view, written language tends to be more carefully planned than spoken language, and this planning may include aspects of prosodic structure which tend to be absent in spontaneous speech.

The contrary position would be to argue that in spite of the fact that spoken language is planned “on the fly,” and may be subject to hesitations, interruptions and restarts, prosodic wellformedness is inherently more or even exclusively part and parcel of the realization of *spoken* language. After all, prosody is intricately tied to articulatory features such as intensity, duration, and pitch, which are an inherent property of spoken utterances, but not of written texts. Above, we have already seen that this view is problematic, as prosodic features have been found to exist in writing. But arguably, the relation of prosody to written texts is an indirect one only: written texts may be read out, either silently or aloud.

In other words, the generality of the phenomenon of Prosodic Parallelism is presently unclear. First, the proponents themselves and Kentner (2015) in his review noted possible counter-examples within the corpus search (see below); second, it is an open and actually debated question whether a prosodic preference such as the one proposed here relates more closely to spoken or to written German^2^.

### Phenomena and data base

For further tests of the Prosodic Parallelism hypothesis in the spoken mode, initially all phrases from the study by Wiese and Speyer (2015) were selected which had shown a frequency of >3000 tokens in the DeReKo database used for the previous study. The list of such phrases is presented in (4)^3^. The last three, (4)h-j, are those for which Prosodic Parallelism could not be shown to hold in the previous study on written German. The complete set of strong nouns listed in (4)j was taken over from the study by Szczepaniak (2010, p. 111/112) and thus constitutes a non-biased corpus of nouns which was not chosen for the purposes of the present study. For further discussion of the list of these nouns, see the section devoted to the strong genitive nouns below. In the case of *(un)gern(e)*, the test does not apply to a phrase consisting of two words, but to a complex word consisting of two morphemes. However, there is evidence that such prefix-stem combinations consist of two phonological words; see, e.g., Wiese (2000). In other words, *(un)gern(e)*, with main stress on the prefix, probably does not differ in prosody from the small phrases provided by the other examples in terms of prosodic structure.

(4) Phrases selected for search
  - die, der/eine, einer + Tür(e) ‘the, a + door, nom./acc. vs. gen./dat.’
  - des/eines + Tag(e)s ‘the/a + day, gen. sg.’
  - des/eines + Jahr(e)s ‘the/a + year, gen. sg.’
  - (un) + gern(e) ‘(not) + gladly’
  - bin, war, ist, sind, seid / waren, seien, werden, wurde, wurden + nah(e) ‘am, was, is, are, are /were, would be, will (sg./pl), were + near’
  - heut(e) + früh/morgen ‘today + early/morning’
  - nah(e) + bin, war, ist, sind, seid / waren, seien, werden, wurde, wurden ‘near + verb’
  - dem/einem + Tag(e) ‘the/a + day, dat. ag.’
  - sehr/richtig + gern(e) ‘very/really + gladly’
  - des/eines ‘the/a’, gen. s.g' + 162 strong monosyllabic nouns: a. masculine: Bach, Berg, Brand, Bund, Darm, Dienst, Feind, Fisch, Flug, Freund, Frost, Gang, Geist, Grund, Hang, Hof, Hund, Kampf, Kauf, Kern, Klang, Koch, Kopf, Krieg, Krug, Lärm, Leib, Lohn, Mond, Mord, Müll, Mut, Ort, Pfahl, Plan, Rang, Rat, Raub, Raum, Ring, Ruf, Rumpf, Saal, Sand, Sarg, Schein, Schirm, Schlag, Schlauch, Sieg, Sinn, Sohn, Spott, Spruch, Staat, Stab, Stahl, Stamm, Stand, Staub, Stein, Stern, Stier, Stock, Stoff, Streit, Strom, Stuhl, Sturm, Tag, Teich, Teil, Text, Tisch, Tod, Traum, Trost, Turm, Wald, Weg, Wein, Wert, Wind, Wirt, Wunsch, Zahn, Zaun, Zoll, Zorn, Zug, Zweck
    b. neuter: Amt, Bad, Bein, Bett, Bier, Bild, Blatt, Blech, Blut, Boot, Brett, Brot, Ding, Dorf, Fach, Feld, Fell, Fett, Fleisch, Geld, Gold, Grab, Haar, Haupt, Heer, Heft, Heil, Heim, Hirn, Hoch, Horn, Huhn, Jahr, Kalb, Kind, Kleid, Korn, Land, Laub, Leid, Licht, Lied, Lob, Loch, Mahl, Meer, Moor, Obst, Ohr, Paar, Pferd, Rad, Rind, Rohr, Schaf, Schiff, Schnitt, Schwein, Seil, Spiel, Stück, Tal, Tier, Tuch, Volk, Weib, Werk, Wohl, Wort, Zelt, Ziel

The restriction to phrases with moderately large frequencies was necessary because, as noted above, corpora of spoken language are generally much less comprehensive than those of written language. For German, the *Database for Spoken German (DGD2*, http://dgd.ids-mannheim.de, see description in Schmidt, 2014*)* has been compiled as a collection of spoken audio files and transcripts. While the *DeReKo* corpus of written German contained about 25 bn. word forms in 2014, the *DGD2* corpus of spoken German contained, at the time of search, about 8.6 million word forms (called ‘tokens’) which were available for search in 4153 different transcripts (The additional audio files contained in the data base are not searchable for lexical items). Thus, the size of the DeReKo corpus of written German is larger by a factor of about 3000. The search was performed over the complete set of corpora of spoken German contained in the DGD data base. It contains corpora from different speaker groups, regional origins (and thus dialectal variants) and registers. It is well-known that different dialects of German show different behavior with respect to the presence of final schwa, and some dialects favor the presence of schwa, while others do not. The present study aims at an over-all picture, and thus includes data from the largest available range of sub-types of spoken German from different varieties, with utterances ranging from reading to storytelling.

The DGD database was searched for the number of relevant cases as listed in (4). Then the Prosodic parallelism hypothesis was tested over contingency tables of the kind illustrated in (2), in order to test whether the number of combinations hypothesized to be preferred is higher than predicted by the given frequencies of their parts. Particular focus is placed on the strong nouns listed in (4)c, see respective section below. The test used for this purpose was Fisher's exact test of independence, with the purpose of verifying that the proportions of frequencies for one variable (presence of schwa) is dependent on the value of some other variable, or alternatively, that the frequencies are independent of each other. This test is usually recommended (as an alternative to the chi-square test or the *G*-test) for small sample sizes, such as the number of items < 5 in one of the cells; see McDonald (2014). In the present case, only one data set (for *dem/einem Tag(e)*) had more than 5 results for all combinations. Therefore, in order to present consistent results, Fisher's exact test is applied to all cases.

## Results

Search items and the *P*-value for this test are summarized in (5), indicating the level of statistical significance. Results with values for *P* < 0.05 are taken as confirmation for the hypothesis. A list of detailed results for all searches performed is presented in Appendix A (Supplementary Material), with absolute numbers for each combination and odds ratio values as an indication of effect size. Results for the set of strong nouns in (4)j are postponed until the following section. Insufficient numbers result from at least one cell with a frequency of zero.

(5) Statistical analysis; Fisher's exact test

**Table:** 

***Search items from* (4)**	***Results for Fisher's exact test***
die, der/eine, einer + Tür(e)	*P* = 0.396, n.s.
des/eines + Tag(e)s	*P* = 0.033^*^
des/eines + Jahr(e)s	Numbers too small, test not applicable
(un) + gern(e)	*P* = 0.000^***^
bin, war, ist, sind, seid / waren, seien, werden, wurde, wurden + nah(e)	Numbers too small, test not applicable
heut(e) + früh/morgen	*P* = 0.041^*^
nah(e) + bin, war, ist, sind, seid / waren, seien, werden, wurde, wurden	Numbers too small, test not applicable
dem/einem + Tag(e)	*P* = 0.027^*^
sehr/wirklich^4^ + gern(e)	*P* = 0.724, n.s.

Non-significant results emerge for the first and the last item used, while for a range of other constructions the hypothesis is either confirmed, or cannot be tested for lack of sufficient data (see details on each item in Appendix A of Supplementary Material). Wiese and Speyer (2015) and Kentner (2015) discuss potential reasons for the failure of the hypothesis for some of the cases studied. Among them is the possibility that determiners (as in *die/der* + *eine/einer*) form a prosodic unit not with the following noun as in *der Tür(e)*, but with the preceding preposition, see discussion above. This would account for the non-significant result in first case given in (5). The failure for the final search item remains unexplained.

### Results for strong genitive nouns

Nouns taking the suffix *-s* for their genitive singular are traditionally called “strong.” The 162 such nouns listed in (4)j constitute a wide range of forms, with strong variation in the appearance of schwa between stems and suffix. Crucially, for present purposes, for these nouns the genitive forms all occur, in principle, with or without schwa preceding genitive ending *-s*, as in *Amt(e)s* ‘office, gen. sg.’ or *Zug(e)s* ‘train, gen. sg’^5^. In their study of written German, Wiese and Speyer (2015) report that across all of these nouns, the Prosodic Parallelism hypothesis is not confirmed, by using the chi-square test. Rather, there is a small but significant trend in the opposite direction. However, a generalized linear mixed model analysis using the lexemes of the data set as a random factor along with the (non-)trochaic noun factor revealed the parallelism preference, once again. This indicates that Prosodic Parallelism may hold here as well^6^. For explanation of the negative result, the authors discuss the fact that a strong lexical bias exists here: some highly frequent nouns are lexicalized with either schwa or no schwa; in particular, for *Jahres* ‘year, gen. sg.’ the schwa-containing variant is much more frequent than the competing form *Jahrs*, independent of context^7^. This is true in spite of the fact that the final consonant /r/ of this noun is one which actually forms a preferred final cluster /r s/.

To illustrate, Table (6) presents the full set of relevant tokens calculated over all 162 nouns, with the number of cases found in the DGD2 database given in the cells of the table, and preferred combinations printed in bold.

(6) Search results for strong nouns

**Table:** 

	**Monosyllabic nouns**	**Bisyllabic nouns**	
des	**35**	754	789
eines	3	**303**	306
	38	1057	1095

As inspection of (6) shows, numbers for these item combinations are too small to make calculations over individual nouns. Frequencies of combinations vary, but the forms preferred according to the Prosodic Parallelism hypothesis (*des* + *monosyllabic* and *eines* + *bisyllabic*) are higher than expected, and vice versa for the non-preferred ones. This trend is significant, with an exact *P* = 0.03 according to Fisher's exact test. The present study of these genitive singular forms of strong nouns thus yields a result which differs from the previous one on written German: Prosodic Parallelism is confirmed for this set of data, even without taking the role of individual lexemes into account.

In summary, for the majority of cases for which there is a sufficiently large amount of data, the independence of the prosodic shapes of adjacent words in a phrase can be refuted. Rather, we find some evidence for such word bigrams to display identical prosodic structures, with two monosyllabic or two bisyllabic constituent parts.

## Discussion

The present corpus-based study of the schwa-zero alternation in German has presented some evidence that prosodic preferences hold in the spoken realization of language. One such preference, Prosodic Parallelism, was tested previously with written language, but it may be the case that the adherence to this preference is stronger in speech than in writing. The twin studies of Wiese and Speyer (2015) and the present one have both demonstrated that the choices of final schwa in two adjacent words are often statistically dependent on each other. In both studies, three and two of the cases studied, respectively, yielded non-significant results, but the majority of cases supported the Prosodic Parallelism Hypothesis. Thus, written and spoken German do not seem to follow fundamentally different preferences in terms of their prosodic organization. It remains to be shown whether this results holds generally. Alternatively, it may be the case that intentions and skills of the writer play a role here: texts intended to be read aloud (and written skillfully for this purpose) might be demonstrated to show the preference to the same (or a higher) degree than spoken, spontaneous, language.

Effects of prosodic parallelism are rather small for many cases in which they could be demonstrated to hold. The reasons for this arise from the fact that other factors intervene, such as lexical preferences for or against the presence of final schwa, the nature of the preceding segment which may even dictate the insertion of schwa as in *Kusses* ‘kiss, gen. sg.’, dialectal preferences, and others. A similar finding emerged from the work by Shih et al. (2015), who studied the two possible forms in the English genitive construction (*the car's wheels* vs. *the wheels of the car*). Their conclusion (Shih et al., 2015, p. 208) is: “rhythm alone does not do or explain everything.” However, in the case of the English genitive construction, there are strong semantic predictors (such as the feature animacy) for the choice, while there do not seem to be such semantic factors for the cases studied here. Furthermore, the present study is not a study of alternating rhythm in general, but of a specific principle, that of Prosodic Parallelism.

The variation in the presence of schwa that clearly exists between different regional variants of German could not be controlled for with the resources presently available, but the dialectal variation which certainly exists in the appearance of schwa does not totally override the preference for prosodic parallelism. If the dialectal variation in the presence or absence of schwa would be the major factor, then Prosodic Parallelism could not be demonstrated, as dialects would prefer either monosyllabic or bisyllabic/trochaic items across the board.

For the spoken corpus studied here, all we can hope is that transcribers followed the spoken form as closely as this is possible in their use of orthographic forms. As pointed out above, in the case of schwa, a faithful rendering of pronunciation by means of orthographic <e> is possible. For written German more generally, the presence of orthographic <e> in the word forms under discussion here may be seen as an instruction (or gentle suggestion?) to the reader that the respective word should be read as a bisyllabic word. In this sense, there is clearly “intended prosody” in the alternative spellings available. We cannot be sure that a particular reader will always follow the advice, but still the suggestion exists.

While this consideration would lead to more regular prosodic phrasing (including prosodic parallelism) for written language, the close relation between speech and prosodic features would yield the opposite result. Another reason for differences between the spoken and written modality in prosodic details may be that prosodic phrases in the spoken modality are shorter than those in the written modality. The reason is that, as mentioned above, planning processes in the spoken modality proceed under more time pressure and with fewer opportunities for revision. In consequence, more re-starts, errors, hesitations, and interruptions influence the flow of speech and thereby the structure of prosodic phrases (Levelt, 1983). In conclusion, written and spoken language may be subject to partially identical and partially different forces as far as adherence to prosodic principles is concerned.

Finally, the reduction of function words may play a role: it has been observed by Hall (1999) and Kentner (2015) that in German many function words (but not lexical words) may optionally be reduced, in terms of vowel shortening or vowel reduction to schwa. This may apply to many of the determiners such as *die* or *eine* listed in (3). Such reduction correlates with a change in foot structure, as in the loss of a foot (defooting). Arguably, such reductions are found more often in the spoken modality than in the realization of written German. However, a written corpus (or one in which spoken German is rendered orthographically) does not allow for a reliable evaluation of such reductions.

## Author contributions

The author confirms being the sole contributor of this work and approved it for publication.

## Funding

Funding for the study reported in this paper was provided by the LOEWE initiative of the state of Hesse, Germany (Fundierung linguistischer Basiskategorien). The sponsor was not involved in any aspect of the study and/or the article.

### Conflict of interest statement

The author declares that the research was conducted in the absence of any commercial or financial relationships that could be construed as a potential conflict of interest.

## Footnotes

^1^“Word-final” is to be taken with a grain of salt. The present study also includes cases in which schwa occurs (or does not occur) between stems and inflectional suffixes, as in the genitive singular forms of nouns in (1)b.

^2^For other languages, there is some evidence for the existence of the same principle. An example is provided by Duanmu (2012) for Chinese: for the majority of constructions of the types [N N] and [V O], the two constituent parts of the phrases are either both monosyllabic or both bisyllabic.

^3^Two phrases were excluded from the initial search for reasons of insufficient frequency (*n* < 3000): *der/guter Hirt(e)* ‘the/good shepherd’, *wär(e) gern(e)* ‘would gladly’.

^4^For the present search, *richtig* ‘truly’ as used by Wiese and Speyer (2015) was replaced by *wirklich* ‘really’ because of the higher frequency of the combination with *gern(e)*.

^5^For what it is worth, the spelling correction system as part of the operating system Mac OS X (for German) marks only one of the resulting 324 forms (162 nouns, each with or without schwa) as incorrect, namely *Mülles* ‘waste, gen. sg.’

^6^With the present set of data, such an analysis is not possible because of the much smaller overall number of cases.

^7^A search in the written DeReKo database (Febr 27, 2016) gives 1,133,209 results for *Jahres*, in contrast to 2957 results for *Jahrs*.
