# Supplementary material for: Prosodic Parallelism—Comparing Spoken and Written Language
Source: Front Psychol. 2016 Oct 19;7:1598. doi: 10.3389/fpsyg.2016.01598 (PMC5069292; doi:10.3389/fpsyg.2016.01598)
Supplement: Supplementary file 1 [file DataSheet1.docx]

# Appendix A

Search results for individual search items given in (3) in absolute numbers; search DGD2 data base April 30, 2016; 24 corpora, 4153 transcripts, 8,641,162 tokens; P-values for Fisher's exact Test and odds ratios

1. die/der, eine/einer Tür(e)

|  | Tür | Türe |
| --- | --- | --- |
| die\|der\|Die\|Der | 483 | 140 |
| eine\|einer\|Eine\|Einer | 29 | 5 |

P=0.396

OR=0.5948

2. des/eines Tag(e)s

|  | Tags | Tages |
| --- | --- | --- |
| des\|Des | 2 | 49 |
| eines\|Eines | 0 | 227 |

P=0.033

OR=237.6337 (assuming null hypothesis)

3. des/eines Jahr(e)s

|  | Jahrs | Jahres |
| --- | --- | --- |
| des\|Des | 0 | 69 |
| eines\|Eines | 0 | 3 |

Test not applicable

4. (un), gern(e)

|  | -- | un\|Un |
| --- | --- | --- |
| gern\|Gern | 2136 | 25 |
| gerne\|Gerne | 2019 | 2 |

P=0.000

OR=0.0846

5. bin|war|ist|sind|seid / waren|seien|werden|wurde|wurden; nah(e)

|  | nah | nahe |
| --- | --- | --- |
| bin\|war\|ist\|sind\|seid | 1 | 6 |
| waren\|seien\|werden\|wurde\|wurden | 0 | 1 |

Test not applicable

6. heut(e), früh/morgen

|  | früh | morgen |
| --- | --- | --- |
| heut | 2 | 90 |
| heute | 17 | 175 |

P=0.041

OR=0.2288

7. nah(e); bin|war|ist|sind|seid / waren|seien|werden|wurde|wurden

|  | nah | nahe |
| --- | --- | --- |
| bin\|war\|ist\|sind\|seid | 5 | 6 |
| waren\|seien\|werden\|wurde\|wurden | 0 | 0 |

Test not applicable

8. dem/einem Tag(e)

|  | Tag | Tage |
| --- | --- | --- |
| dem\|Dem | 86 | 18 |
| einem\|Einem | 121 | 10 |

P=0.027

OR=0.3949

9. sehr/wirklich gern(e)

|  | gern | gerne |
| --- | --- | --- |
| sehr\|Sehr | 139 | 147 |
| wirklich\|Wirklich | 3 | 5 |

P=0.724

OR=1.576
